# *In vivo* CAR-T for the treatment of autoimmune diseases: Current progress and future directions

**DOI:** 10.1016/j.gendis.2026.102245

**Published:** 2026-05-19

**Authors:** Yufan Zhao, Yuhang Liu, Xiaochun Peng, Xin Ma, Quanjun Yang

**Affiliations:** a Department of Pharmacy, Shanghai Sixth People's Hospital Affiliated Shanghai Jiao Tong University School of Medicine, Shanghai 200233, China; b Department of Orthopaedics, Shanghai Sixth People's Hospital Affiliated Shanghai Jiao Tong University School of Medicine, Shanghai 200233, China

**Keywords:** Autoimmune diseases, Immunotherapy, *In vivo* CAR-T, mRNA-LNP, Viral vectors

## Abstract

Autoimmune diseases are a clinical challenge because current immunosuppressive therapies fail to eliminate pathogenic immunocytes, such as autoreactive B cells, plasma cells, and antigen-presenting cells, which induce chronic inflammation. Chimeric antigen receptor T (CAR-T) cell therapy offers a targeted strategy to eliminate these disease-driving populations. Early *ex vivo* CAR-T cell studies on severe autoimmunity have shown effective B-cell depletion and sustained remission. However, its toxicity, cost, and lengthy manufacturing process limit its widespread clinical use. *In vivo* CAR-T technologies—using viral vectors or mRNA lipid nanoparticles to generate CAR-T cells directly within the patient—aim to overcome these barriers. Preclinical and emerging clinical data indicate that these platforms can rapidly reprogram circulating T cells, induce potent B-cell clearance, and achieve clinical improvement in refractory diseases. Viral vectors provide durable expression but raise integration and immunogenicity concerns, whereas mRNA–LNP systems enable transient, controllable CAR expression with greater manufacturing scalability. These advances have positioned *in vivo* CAR-T cells as a promising therapeutic strategy for autoimmune diseases. Continuing progress in delivery specificity, safety engineering, trial design, and cost-effective production is essential for translating this approach into widely accessible treatments. In this review, we synthesized preclinical and emerging clinical evidence, compared delivery platforms, and proposed practical recommendations for safety engineering, trial design, and manufacturing, which will help guide future translational efforts and accelerate clinical adoption.

## Introduction

Autoimmune diseases represent a class of complex chronic inflammatory conditions characterized by the breakdown of immune tolerance. Autoimmune diseases encompass systemic lupus erythematosus, rheumatoid arthritis, and multiple sclerosis, which lead to persistent inflammation and gradual organ damage.^1^^,^^2^ Although glucocorticoids, conventional immunosuppressants, and targeted biological agents have achieved certain efficacy, many patients with systemic autoimmune diseases fail to achieve sustained remission.^3^^,^^4^ Current therapeutic approaches typically suppress the immune system, exposing patients to the risks of infections, malignancies, metabolic toxicity, and cumulative organ damage, while neglecting the basic pathological factors of diseases. Consequently, refractory diseases often occur in a significant proportion of patients, which calls for long-term escalating immunosuppressive therapy.^5^ These limitations highlight the urgent need for more precise therapies that can improve safety and controllability, and substantially shorten the treatment cycle.^6^

At the mechanistic level, autoreactive B cells, long-lived plasma cells, and dysfunctional antigen-presenting cells, particularly plasma cell-like and traditional dendritic cells, perpetuate chronic autoimmunity by continuously producing autoantibodies, activating complements, and persistently stimulating T cells.^7^ Elimination therapies targeting B cells, such as rituximab and anti-B-cell activating factor (BAFF) antibodies, provide significant proof-of-concept for the elimination of pathogenic B cell lineages and reestablishment of the dysregulated immune circuit. However, these therapies often fail to eliminate existing or long-lived self-reactive clones within the tissues, resulting in disease recurrence in many patients. Common side effects include increased risk of infection, development of drug tolerance, and potential toxicity.^8^^,^^9^ Consequently, there has been a significant focus on the study of new anti-B-cell therapies in recent years.

In recent years, immune system resetting has emerged as a promising therapeutic strategy. Chimeric antigen receptor (CAR) T-cell technology provides a fundamentally different, antigen-directed approach: a synthetic receptor couple-specific antigen recognition (usually via an scFv) to intracellular activation and costimulatory modules so that T cells can kill target cells in a major histocompatibility complex (MHC)-independent manner.^10^^,^^11^ Successful clinical application of CAR-T cells targeting cluster of differentiation 19 (CD19) in B-cell malignancies has demonstrated that genetically engineered cytotoxic lymphocytes can effectively eliminate pathogenic autoreactive B cells. These findings have generated interest in the application of CAR-T technology in autoimmune diseases, in which autoreactive B cells and plasma cells play a critical role in the persistence of autoantibodies and chronic T-cell activation. Notably, early clinical trials utilizing *ex vivo*-produced CD19 CAR-T cells for refractory systemic lupus erythematosus, inflammatory myopathy, and systemic sclerosis have shown significant efficacy, including rapid B-cell reduction, normalization of serum markers, and a substantial number of patients achieving remission without the need for medication.^12^, ^13^, ^14^ Importantly, expanding the target landscape in autoimmune indications requires moving beyond lineage-wide depletion toward strategies that enhance antigen precision and circuit-level control. Antigen-specific approaches, such as chimeric autoantibody receptor T cells (CAAR-T) that present disease-relevant autoantigens to selectively eliminate pathogenic B-cell clones, or peptide–MHC-directed CAR platforms capable of recognizing autoreactive T cells or pathogenic antigen-presenting complexes, illustrate how engineered cells can discriminate disease-driving immune populations from protective immunity.^6^^,^^15^ Complementing clonal precision, targeting upstream immune regulators, such as the CD40–CD40L axis or B-cell survival pathways (BAFF/APRIL), offers a systems-level strategy to interrupt pathogenic immune amplification loops rather than simply removing terminal effector cells.^16^ Moreover, CAR innovations increasingly leverage logic-gated and context-aware circuits to enhance specificity and safety beyond single-antigen targeting. Logic gating can integrate multiple inputs, such as dual antigens, cytokine landscapes, or microenvironmental cues, to restrict effector activation to pathological contexts. This approach mitigates off-target toxicity and expands the repertoire of feasible targets. Nolan-Stevaux and Smith outline Boolean and contextual logic frameworks in engineered I cells, which discriminate complex antigen combinations and tissue states, thereby enhancing precision control. Upadhyay et al further emphasize the importance of cytokine-guided logic layers that program recognition, safety, and persistence, enabling adaptive and programmable CAR responses tailored to specific disease environments.^17^^,^^18^

The traditional process of preparing autologous CAR-T cells is costly, slow, and technologically complex, necessitating individualized collection of cells, viral transduction, amplification, and release testing, which can take several weeks.^19^^,^^20^ Furthermore, considering its broad application in non-malignant chronic diseases, risks such as cytokine release syndrome and neurotoxicity remain significant concerns.^21^^,^^22^ Several parallel innovations have emerged to broaden their applicability to autoimmunity. Antigen-specific precision strategies, such as CAAR-T, selectively recognize pathogenic B-cell receptors and can spare protective B-cell clones, offering a way to treat autoantibody-mediated diseases with reduced collateral depletion. CAAR-T was established in proof-of-concept studies on pemphigus vulgaris.^6^^,^^23^ Regulatory-phenotype CAR-T cells (CAR-Tregs) aim to deliver targeted immunosuppression to inflamed tissues while preserving systemic immunity, making them particularly attractive for organ-specific or tolerance-restoration strategies.^24^ Allogeneic “off-the-shelf” CAR-T products and genome-editing approaches seek to overcome production and access barriers by creating universal donor cells, albeit with challenges of host-versus-graft biology that gene editing can partially mitigate.^25^ These challenges have sparked considerable interest in *in vivo* CAR-T technology, which employs viral vectors or lipid nanoparticles to directly deliver CAR gene materials into patient T cells, thereby facilitating the on-site generation of CAR-T cells. This *in vivo* strategy circumvents *ex vivo* operational steps, thereby reducing the production time from several weeks to a few hours or days, lowering manufacturing costs, and potentially allowing adjustable or transient CAR expression. This approach offers greater control than permanently edited *ex vivo*, thereby enhancing safety.^26^, ^27^, ^28^

These advancements collectively render *in vivo* CAR-T therapy a transformative treatment strategy, capable of achieving precise targeted immune modulation, facilitated by straightforward manufacturing processes and the potential for a curative immune system “reset”. As clinical experience accumulates, *in vivo* CAR-T therapy is expected to overcome the long-standing limitations of traditional immunosuppressive therapies and reshape the treatment landscape of severe autoimmune diseases.

## Challenges of traditional *ex vivo* CAR-T therapy for the treatment of autoimmune diseases

Although CAR-T cell therapy has demonstrated significant efficacy in treating B-cell malignancies, its application in chronic autoimmune diseases encounters several fundamental challenges, including toxicity, manufacturing complexity, cost, scalability, and biological limitations (Fig. 1). Collectively, these issues hinder the widespread adoption of current *ex vivo* CAR-T platforms and necessitate the development of *in vivo* CAR-T strategies to streamline the production process and improve safety.

**Figure 1:**
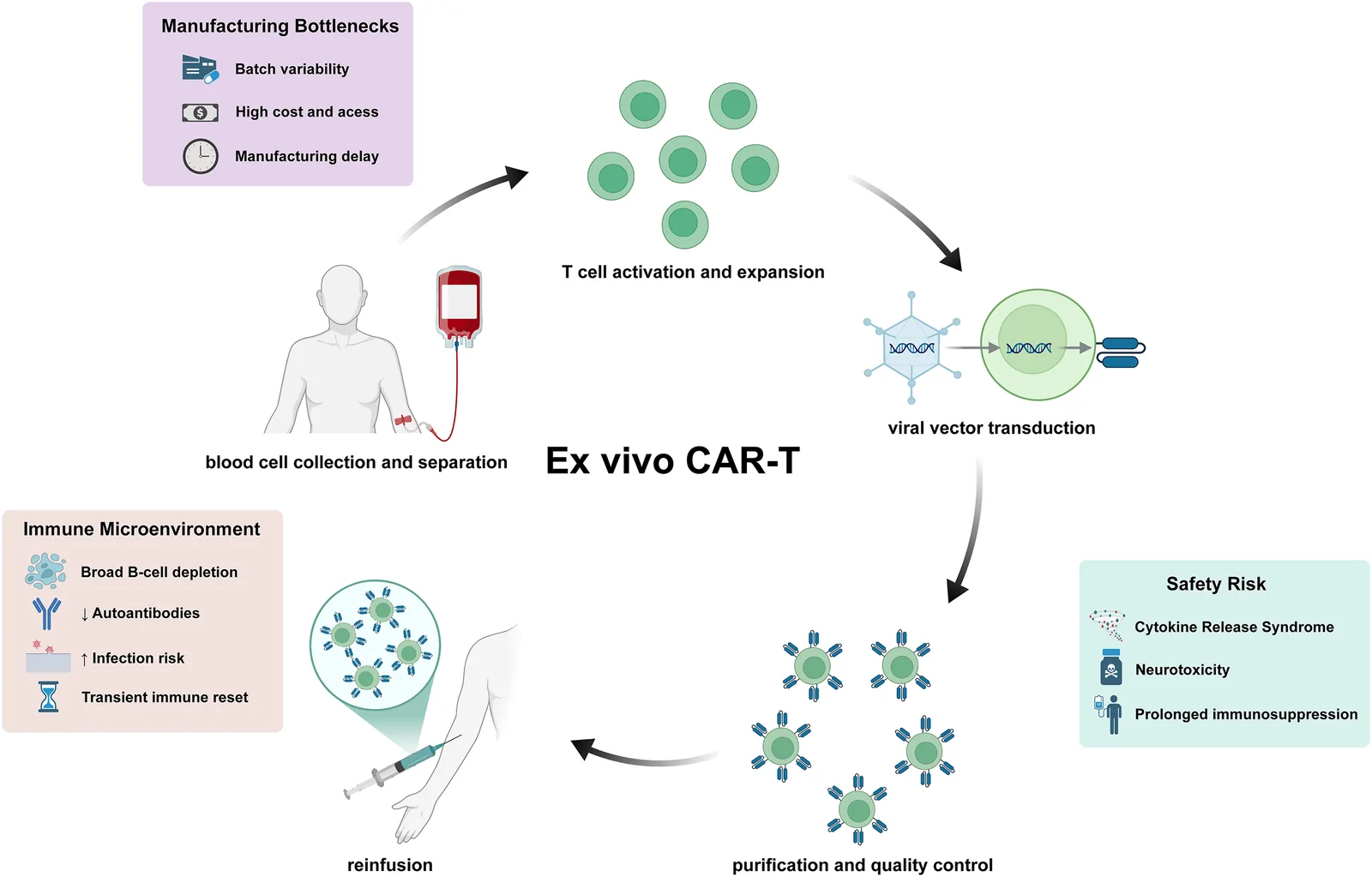
Conventional *ex vivo* CAR-T manufacturing.

The primary challenge in extending *ex vivo* CAR-T therapy is the risk of severe inflammatory toxic reactions, including cytokine release syndrome and immune effector cell-associated neurotoxicity syndrome. Cytokine release syndrome results from the rapid activation of CAR-T cells and release of elevated cytokine levels (IL-6, IL-1, IFN-γ, and GM-CSF), which can lead to high fever, vasodilation, capillary leakage, coagulation dysfunction, and multiple organ dysfunction, necessitating active supportive treatment. Immune effector cell-associated neurotoxicity syndrome is characterized by symptoms such as encephalopathy, aphasia, convulsions, and, in rare instances, cerebral edema, which may occur concurrently or independently of cytokine release syndrome. Despite the development of management strategies (tocilizumab, corticosteroids, and IL-1 inhibitors) that have improved treatment outcomes, the unpredictable severity of these toxic reactions renders traditional CAR-T therapy unsuitable for patients with stable chronic autoimmune diseases, particularly for those without life-threatening disease activity. These safety concerns have been consistently highlighted in clinical studies and expert consensus guidelines, establishing toxicity as the principal barrier to the application of CAR-T therapy beyond cancer treatment.^29^, ^30^, ^31^

There are biological limitations associated with *ex vivo* CAR-T cell treatment, particularly in autoimmune diseases. First, clinical experience with CD19-directed CAR-T cells has revealed that antigen escape is a recognized mechanism contributing to disease recurrence, which may also hinder persistent clearance of autoreactive B-cell clones.^32^ Second, long-lived plasma cells located in the bone marrow and inflammatory lesions have demonstrated resistance or heterogeneous phenotypes to conventional CD19-directed strategies in numerous studies, potentially leading to the continuous production of pathogenic autoantibodies.^33^ Additionally, tissue-resident memory B cells and ectopic lymphoid structures, such as those found in systemic lupus erythematosus, rheumatoid arthritis, and multiple sclerosis, can act as reservoirs for localized B cell responses. In certain tissues, the microenvironment and physical barriers may impede the delivery and penetration of CAR-T cells, limiting their ability to clear these reservoirs.^34^ Furthermore, the manufacturing process of *ex vivo* activation and expansion can result in T cell exhaustion, thereby reducing persistence and weakening long-term immune reprogramming.^35^ These biological challenges suggest that traditional CAR-T cell therapy is more effectively optimized for hematological malignancies than for chronic locally occurring autoimmune diseases.

CD19 and B-cell maturation antigen (BCMA) are the two primary targets utilized in autoimmune CAR-T cell research as they are expressed across a diverse array of B-cell populations. This broad expression pattern implies that CAR-T cells can eliminate both pathogenic and protective B cells. Although a significant reduction in B cells may be acceptable in the context of malignant tumors, it presents substantial challenges for autoimmune patients who require lifelong treatment. These challenges include long-term hypogammaglobulinemia, recurrent infections, and dependence on intravenous immunoglobulin. Long-term follow-up studies of patients undergoing tumor CAR-T receptor transplantation have indicated that immune reconstitution can take several years and may not reach completion.^36^^,^^37^ This observation suggests that applying similar methods to large-scale autoimmune populations could lead to persistent immune deficiencies, which is a critical issue that warrants careful consideration. These concerns underscore the necessity of developing strategies that enable short-term adjustable B-cell targeting, which represents a significant theoretical advantage of the *in vivo* CAR-T platform.^26^

Autologous CAR-T cell therapy requires a complex, multistep, and personalized manufacturing process. This process includes blood cell collection and separation, cryopreservation and transportation, T-cell activation and expansion, viral vector transduction, purification and quality control, and final reinfusion. Typically, the “vein-to-vein” process takes several weeks and may be delayed or even fail due to factors such as low-quality initial collection, inadequate T cell yield, failure of *ex vivo* expansion, poor quality control, or logistical obstacles. This protracted production timeline presents a significant barrier to the use of this therapy in autoimmune diseases.^20^^,^^38^

Moreover, the current cost of autologous CAR-T cell therapy is prohibitively high for chronic diseases, limiting its widespread dissemination. The procurement cost for commercial CAR-T products typically ranges from USD 373,000 to USD 475,000. When considering the costs associated with hospitalization, complication management (such as the handling of cytokine release syndrome/immune effector cell-associated neurotoxicity syndrome), bridging treatment, testing, and related medical resources, the total treatment cost can exceed USD 500,000, or even USD 1,000,000.^39^^,^^40^ Although these expenditures may be acceptable in the context of treating refractory hematological malignancies, they are not feasible for long-term management of non-fatal autoimmune diseases that affect millions of patients globally. Consequently, affordability remains a primary obstacle for the application of autologous CAR-T cell therapy in non-tumor fields.^41^^,^^42^

## New technical platform of *in vivo* CAR-T cells for the treatment of autoimmune diseases

*In vivo* CAR-T approaches seek to bypass *ex vivo* cell manufacturing by delivering CAR-encoding genetic cargo directly to a patient's lymphocytes, thereby producing CAR-expressing T-cells on-site. These strategies fall into two broad classes: viral vector-based platforms, which can provide durable expression through genomic integration or long-lived episomal persistence, and nonviral nanoparticle platforms, notably ionizable lipid nanoparticles carrying modified mRNA, which produce transient, controllable CAR expression with no genomic integration (Fig. 2).

**Figure 2:**
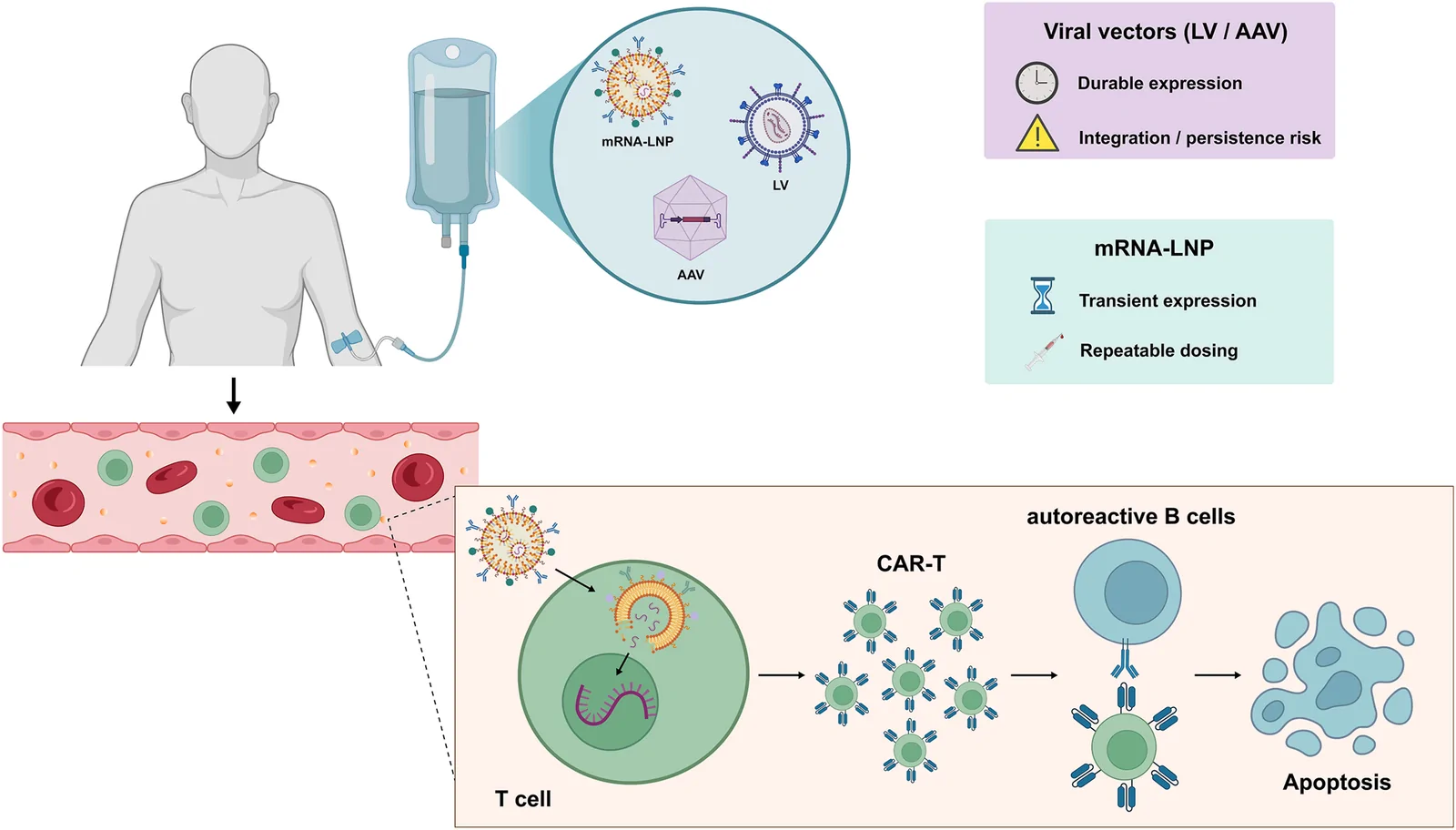
*In vivo* CAR-T strategies deliver genetic cargo directly to endogenous T cells via systemic or targeted administration, enabling rapid on-site CAR generation with manufacturability and safety advantages over *ex vivo* approaches.

Although platform-specific trade-offs remain, the growing body of animal and early human evidence supports cautious clinical translation and motivates platform selection that matches disease biology and safety profiles (Table 1).

**Table 1:** Comparison of *in vivo* CAR delivery platforms for the treatment of autoimmune diseases.

Platform	Expression duration	Genomic integration	Immunogenicity	Payload capacity	T-cell targeting specificity	Translational status	Key safety risks	Mitigation strategies	Reference
Lentiviral vectors (LV)	Durable, weeks to months	Integrating	Moderate	∼8–10 kb	Achievable via surface engineering	Extensive preclinical and NHP data; early translational/IND-stage programs	Genomic integration → insertional mutagenesis; off-target transduction; prolonged CAR persistence; cytokine release syndrome	SIN-LV design; integration-site mapping; T-cell–selective targeting ligands; dose control; suicide switches	48,49,53
Adeno-associated viruses (AAV)	Long-lasting, dependent on cell turnover	Predominantly non-integrating	Moderate to high (pre-existing neutralizing antibodies common)	∼4.7 kb	Limited; requires capsid or promoter engineering	Robust preclinical evidence; limited peer-reviewed human *in vivo* CAR data	Pre-existing neutralizing antibodies; liver tropism/off-target expression; limited payload; dose-related toxicity	Capsid engineering; immune modulation; tissue-specific promoters; dual-AAV/split cassettes	44,69
Lipid nanoparticles (LNP)	Transient, hours to days; repeat dosing feasible	mRNA non-integrating	Low to moderate	∼1–2 kb (CAR mRNA)	via antibody or ligand targeting	Strong preclinical data; first peer-reviewed human autoimmune *in vivo* CAR report	Innate immune activation; anti-PEG/LNP antibodies; hepatic uptake; limited persistence	Modified mRNA chemistry; ionizable lipid optimization; Ab- or ligand-targeted LNPs; dose	27,59,67

### Viral vectors for *in vivo* CAR-T cell therapy

*In vivo* CAR-T therapy positions the viral vector as a central component of the delivery strategy owing to its high transduction efficiency, prolonged expression, and extensive clinical application and production experience in gene therapy. Previous reviews and opinion articles have highlighted that lentiviral vectors (LV) and adeno-associated viruses (AAV) are the most established tools for achieving stable CAR expression in T cells following systemic or targeted administration, thereby ensuring stable and long-term expression.^43^^,^^44^ Concurrently, novel virus-derived envelopes or virus-like delivery vectors (EDV/VLP) enhance modular targeting and formulation flexibility, potentially expediting the translation process.^45^ These approaches are anticipated to simplify the production process and increase accessibility, offering advantages over *ex vivo* workflow. However, each vector category presents distinct trade-offs concerning affinity, formulation capacity, immunogenicity, and safety, which influence its suitability and safety for autoimmune indications. At present, the majority of *in vivo* CAR-T strategies are in preclinical or early clinical stages.^26^

*In vivo* and *ex vivo* engineering of CAR-T cells using the LV platform has advanced rapidly in both preclinical and translational research. This progress is primarily due to the chromosomal integration capabilities of lentiviruses, which facilitate sustained CAR expression in proliferating lymphocytes, thereby promoting long-term efficacy and cell persistence.^43^ Targeted lentiviruses, such as CD4-LV and CD8-LV, have demonstrated selective *in vivo* labeling and generation of functional CAR-T cells in various humanized mouse models, exhibiting robust antigen-dependent cytotoxicity.^46^^,^^47^ Building on this foundation, surface-engineered lentiviral particles, including the VivoVec series and UB-VV100, incorporate T cell-targeting ligands and co-stimulatory signals on their surfaces to enhance the selectivity and functional expansion of CAR-T cells generated *in vivo*. Preclinical reports have indicated that CAR is effectively generated in small animal models, achieving persistent B-cell clearance of target cells.^48^ Notably, compelling translational evidence has emerged from nonhuman primate studies using surface-engineered lentiviral particles. In macaques, VVVec particles (VVPs) displaying an anti-CD3-based multidomain fusion ligand enabled efficient *in vivo* T-cell transduction without lymphodepletion, generating robust CAR-expressing T-cell populations that expanded rapidly in the circulation and induced near-complete B-cell depletion within one week. B cell clearance was durable, persisting for up to 8–11 weeks in the responding animals, and CAR-T cells re-expanded upon B cell recovery, indicating functional persistence. A clear dose–response relationship was observed, as lower vector exposure failed to achieve sustained CAR expression or B-cell depletion. Together, these findings demonstrate potent on–target activity with acceptable short-term tolerability and establish a critical translational bridge for first-in-human *in vivo* lentiviral CAR-T studies.^49^

AAV was one of the earliest viral vectors employed for the *in vivo* delivery of CAR genes owing to its low pathogenicity, well-defined safety profile, and capacity to facilitate long-term epigenetic and externally introduced gene expression in non-proliferating cells, such as those found in the liver, muscle, and nerve tissues.^50^ In a notable proof-of-concept study by Wu et al, a single intravenous dose of an AAV vector encoding a CD4-directed CAR administered to humanized NCG-HuPBL mice bearing luciferase-tagged MT-2 adult T-cell leukemia xenografts produced detectable CAR expression on CD45^+^ cells within 48 h and induced marked tumor regression with a clear survival benefit compared with controls. The authors observed durable CAR detection across lymphoid tissues and only modest cytokine elevation during CAR expansion, supporting the feasibility of a one-time *in vivo* reprogramming approach while underscoring the need for dose-finding and biodistribution studies.^44^ However, AAV is not without limitations; it has restricted packaging capacity, pre-existing anti-AAV immune responses in humans, and host immune responses directed against both the capsid and transgenes. These factors may hinder its efficacy and biodistribution.^51^^,^^52^ Consequently, while AAV-mediated *in vivo* CAR production is functionally viable and highly appealing, its clinical applicability remains contingent upon further engineering and clinical validation to address the risks associated with carrier capacity, immunogenicity, dosage, and biodistribution.^50^

Safety, biodistribution, and immune-mediated limitations are critical factors in selecting vectors that significantly influence the delivery route and treatment design for autoimmune diseases. Historical reports on the insertional carcinogenicity of integrated retroviral vectors in hematopoietic gene therapy have underscored the importance of vector integration site characteristics and selection of promoters and enhancers.^53^ The immunogenicity of the virus, along with its innate immune response, may limit its transduction efficiency, alter its biodistribution, or induce systemic inflammation. Consequently, when employing *in vivo* viral delivery for patients with immune system disorders, careful dose determination, capsid and particle engineering, and immune management strategies are essential.^54^ Regulatory agencies typically mandate rigorous non-clinical and clinical evaluations of vector biodistribution, transgene spread, deleterious transduction, and genetic toxicity, particularly when systemic administration is used to treat autoimmune diseases characterized by widely expressed antigens. These requirements are stringent and may influence the development pathway and trial design.^55^

*In vivo* CAR-T delivery platforms utilizing viral vectors have shown considerable efficacy and functional transfer capabilities in preclinical trials by targeting specific vectors and engineered particles. Increasing evidence from non-human primate model conversions supports the feasibility and controllability of this technology for generating durable functional CAR-T cells *in vivo*. However, several engineering improvement plans are necessary to address the immunogenicity, specificity, and payload limitations. These advancements have led us to make specific choices for different diseases; for instance, a systemic lentiviral vector or enveloped delivery vehicle (EDV) platform is employed for B-cell-mediated autoimmune diseases (such as systemic lupus erythematosus), whereas a more localized AAV method is utilized for organ-specific autoimmune diseases. Furthermore, there is potential for combined strategies, such as concurrent delivery of immunomodulatory agents.

## mRNA–LNP platform for *in vivo* CAR-T cell therapy

Lipid nanoparticles (LNP) have emerged as the primary non-viral platform for *in vivo* mRNA delivery, owing to their advanced clinical development, scalability for mass production, and ability to encapsulate and protect chemically modified mRNA, thereby facilitating efficient cytoplasmic translation. Typically, LNP consist of ionizable cationic lipids, auxiliary lipids, cholesterol, and glycosylated lipids, which enable liposomes to escape the endoplasmic reticulum and achieve transient protein expression.^56^^,^^57^ These attributes are particularly appealing for applications requiring adjustable short-term CAR expression, striking a balance between efficacy and safety in nonmalignant diseases.^58^ Ongoing basic research on the design and transformation of LNPs, along with pertinent clinical experience, has elucidated these characteristics and clinical insights gained from mRNA vaccines and therapeutic drugs.

Rurik et al demonstrated that LNP could effectively generate functional CAR-T cells *in vivo*. The results of their conceptual validation were highly convincing. They delivered modified mRNA encapsulated in lipid nanoparticles that specifically targeted T cells, thereby producing short-lived anti-fibrotic CAR-T cells. These cells could alleviate cardiac fibrosis in mouse models.^59^ This study established the biological feasibility of reprogramming circulating T-cells *in vivo* and provided a practical framework for antigen selection, targeting ligand strategies, and systemic LNP dosing considerations. Subsequently, Mitchell optimized the chemical structures of ionizable lipids and antibody-linked lipid nanoparticles (Ab-LNPs), enhancing their extravascular targeting and ability to selectively transfect circulating T cells, resulting in dose-dependent CAR expression and significant B-cell clearance effects in mouse models.^27^ Furthermore, prior research indicated that by screening and optimizing ionizable lipids, LNP can efficiently deliver mRNA to primary human T cells under *ex vivo* conditions and induce functional protein expression, thereby establishing a crucial technical foundation for the development of *in vivo* CAR-T cell platforms.^60^ Bimbo et al demonstrated T cell-specific non-viral DNA delivery using targeted lipid nanoparticles to generate CAR-expressing T cells *in vivo*. In preclinical models, this approach resulted in durable CAR expression and antigen-specific target cell depletion, demonstrating acceptable short-term tolerability. This work expands the options for non-viral LNP cargo beyond mRNA, paving the way for longer-lasting *in vivo* CAR-T therapies.^61^ Liu et al reported the generation of CAR-T cells *in vivo* for the treatment of myocardial fibrosis, revealing that *in situ* engineered CAR-T cells were trafficked to the injured myocardium and reduced the fibrotic burden in animal models. This finding supports the platform's applicability to organ-specific and non-oncologic indications.^62^

Mechanistically, two complementary engineering strategies were used in this field. The first involves achieving passive optimization through the adjustment of formulation and lipid selection, which facilitates the biodistribution of liposomes with an enhanced extrahepatic tendency towards immune organs. This can be accomplished by introducing selective organ targeting (SORT) molecules or altering internal/external charges to improve enrichment in the spleen and other secondary lymphoid organs.^63^ The second strategy involves decorating the surface of LNPs with antibodies, nanobodies, or ligands that target pan-T molecules (CD3, CD5, and CD8) to promote selective binding and internalization by T cells.^64^ These strategies have been demonstrated to effectively deliver functional CAR mRNA to T cells *ex vivo*, thereby inducing surface CAR expression. Substantial evidence supports this hypothesis for *ex vivo* application of primary human T cells. Notably, CAR expression is typically transient, peaking within a few hours to 24–48 h before declining over several days. This kinetic profile is advantageous because it minimizes the risk of long-term target-related immunosuppression or irreversible toxicity and allows repeated administration when necessary.^59^

In addition to its feasibility, *in vivo* CAR-T cell therapy utilizing LNP presents several practical advantages for the treatment of autoimmune diseases. First, the manufacturing process of LNP is compatible with centralized Good Manufacturing Practice (GMP) production and can be streamlined through freeze-drying and formulation methods.^65^^,^^66^ Importantly, the absence of gene integration eliminates the risk of insertional mutations associated with the integrated viral vectors. In the context of autoimmune diseases, the transient expression of CARs may be more advantageous, facilitating immune “resetting” while avoiding the permanent depletion of protective cell populations. Furthermore, preclinical safety studies have investigated the simultaneous delivery of regulatory RNA, such as small interfering RNA, aimed at temporarily inhibiting PD-1 to enhance the efficacy of CAR-T cells while minimizing systemic immune activation.

Theoretical and preclinical advantages have now begun to translate into clinical trial experience involving human patients. A recent report described an *in vivo* LNP–mRNA approach that successfully generated T cells expressing CD19 CAR in patients with refractory systemic lupus erythematosus, leading to a rapid and significant reduction in B cells and an improvement in the condition of treated patients. This study utilized an LNP drug carrier for T cells, developed by a consortium of enterprises and academic institutions. Although this report is still in its preliminary stages and has a limited scale, it is the first to demonstrate that LNP–mRNA-mediated *in vivo* CAR generation is safe and biologically active against human autoimmune diseases. This clinical milestone supports the accelerated evaluation of the LNP platform in other refractory autoimmune diseases and underscores the necessity for stringent drug monitoring and standardized biomarker endpoints, including CAR cell frequency, anti-drug immune response, and humoral reconstitution.^67^

In conclusion, the mRNA–LNP platform represents the foremost nonviral approach for achieving clinically scalable *in vivo* CAR-T cell generation. The distinctive advantages of LNP–mRNA include its capacity for transient expression, adjustable CAR expression, lack of gene integration, and ease of manufacture, rendering it highly appealing for the treatment of autoimmune diseases. The safety and ability to modulate treatment dosage are critical factors in managing such conditions. Current preclinical models, along with an initial series of human cases, provide compelling evidence for advancing meticulously controlled clinical trials that target specific diseases. Concurrently, it is essential to consider the requirements for biodistribution, innate immune response, immunogenicity associated with repeated dosing, and standardized immune monitoring.

## Future outlook of *in vivo* CAR-T cell therapy for the treatment of autoimmune diseases

Future translation of *in vivo* CAR-T therapy for autoimmune diseases will depend on the field's ability to integrate biological precision with engineering control. Rather than relying on broad immune depletion, next-generation strategies must increasingly focus on selective immune modulation with antigen-specific and logic-gated designs that distinguish pathogens from protective immune compartments. In parallel, delivery platforms will need to evolve toward systems that provide efficient yet tightly regulated CAR expression, minimizing off-target effects and long-term genomic risk, while improving tissue access to sites of active inflammation, such as bone marrow, joints, and the central nervous system. Equally important is the development of safe architectures that enable dynamic control over CAR-T persistence and function, including transient expression systems, built-in termination switches, and immune-evasive designs. These advances must be matched by progress in clinical implementation, including standardized production, scalable manufacturing, and rational patient stratification (Fig. 3).

**Figure 3:**
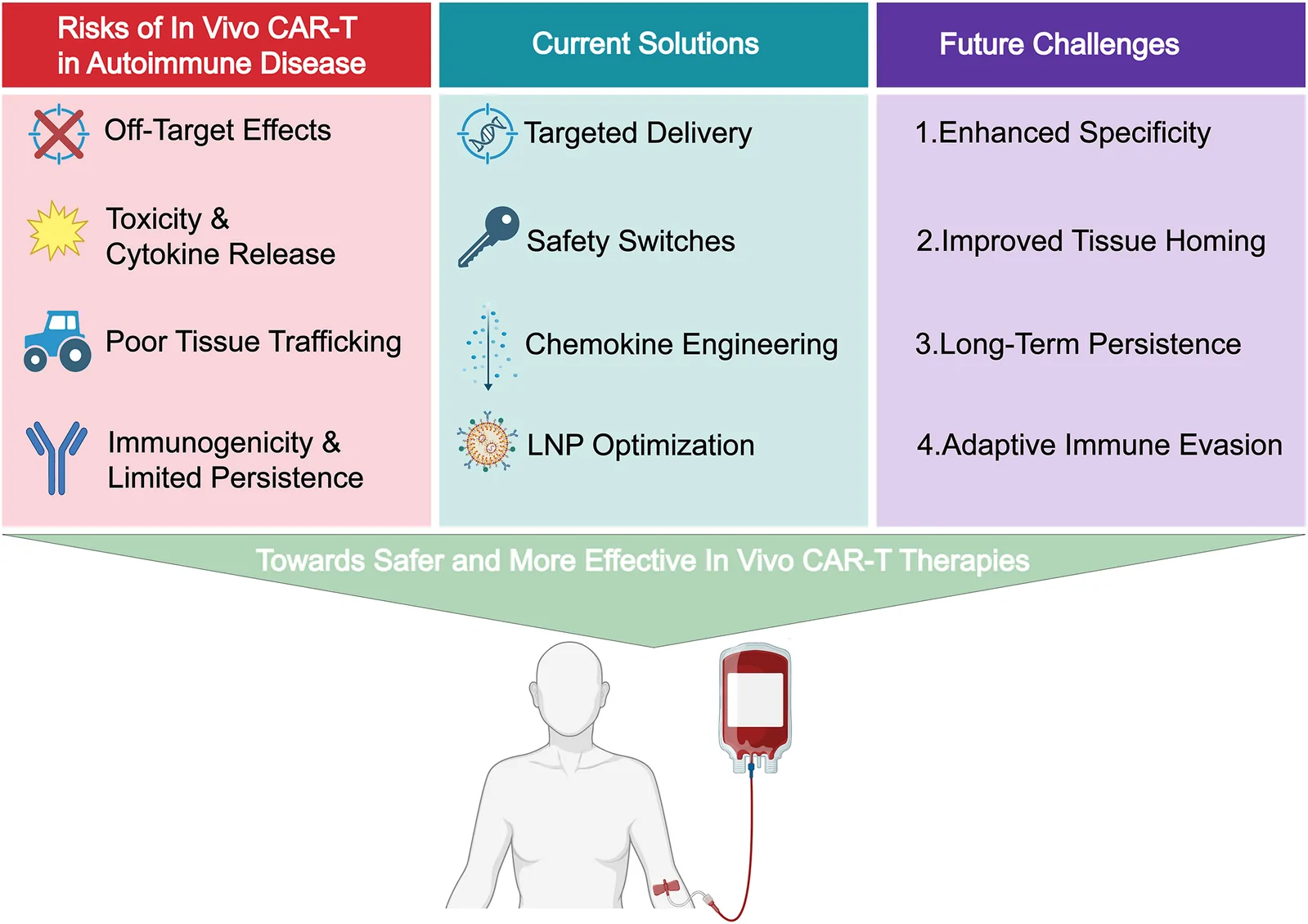
*In vivo* CAR-T risks and future.

## Reducing biological and immune risks of *in vivo* CAR-T therapy

The primary challenge in transforming *in vivo* CAR-T therapy into first-line conventional treatment for autoimmune diseases is to minimize the risks of uncontrolled systemic inflammation and neurotoxicity associated with the *ex vivo* CAR-T method. Given that these symptoms can be life-threatening, careful consideration must be given to the selection of vectors and doses for nonmalignant indications.^68^ Employing non-integrating transient expression platforms, such as mRNA–LNPs or non-integrating EDVs, can significantly mitigate the long-term genetic toxicity risk compared with integrating vectors. Additionally, targeted delivery methods can help limit non-targeted transduction and liver uptake, thereby avoiding potential adverse inflammatory reactions.^56^^,^^59^ Optimizing nucleic acid chemistry and immunomodulatory adjuvants are essential to reduce innate immune perception and cytokine induction following administration. These strategies are pivotal for the rapid clinical translation of therapeutic mRNA and play a central role in safe repeated administration in autoimmune populations.^27^^,^^60^ In cases where integrated viral platforms are used and persistent CAR expression is necessary, stringent vector design, comprehensive insertion site monitoring, and long-term tracking must be implemented to mitigate the historical risk of insertional carcinogenesis, as indicated in early gene therapy trials.^53^^,^^69^ Regulatory frameworks and related guidance documents are currently being developed for Investigational New Drug (IND) strategies, with clear expectations for biodistribution, toxicology, and monitoring. Developers must adhere to these requirements before initiating the first human trials for autoimmune diseases.

## Clinical translation and early trial design

A critical translational gap for *in vivo* CAR-T therapy is whether CAR-expressing T cells generated in the circulation can efficiently traffic to and function within disease-relevant tissue compartments, such as the bone marrow, central nervous system, and inflamed joints. Access to these niches is governed by chemokine gradients, endothelial adhesion programs, and anatomical barriers, including the blood–brain barrier.^70^ Nevertheless, both clinical and preclinical evidence indicate that CAR-T cells can reach sanctuary sites under appropriate conditions; adoptively transferred CAR-T cells have demonstrated activity in central nervous system lymphomas, and LNP-generated CAR-T cells have been shown to home to the injured myocardium and exert local therapeutic effects in fibrosis models.^59^^,^^70^ Engineering strategies, such as chemokine receptor modulation, adhesion molecule tuning, and organ-targeted delivery platforms (including extrahepatic and spleen-biased LNP formulations), offer tractable routes to improve tissue-specific homing and persistence.^71^, ^72^, ^73^, ^74^ Recent studies in mice/models have shown that fusogenic nanocapsules/targeted LNP delivery systems for T cells can generate functional CAR-T cells *in vivo*, and in the collagen-induced arthritis model, they lead to B cell depletion and significantly improve the outcome of arthritis.^75^ From a translational standpoint, early clinical studies should incorporate tissue-level biodistribution and functional readouts, including paired blood–tissue sampling, CAR copy-number kinetics in target organs, and imaging-based cell tracking to determine whether *in vivo*-generated CAR-T cells achieve sufficient exposure and activity at disease sites.^76^

Early clinical development should prioritize patients with severe and refractory autoimmune diseases. When the potential benefits outweigh the higher initial risks, a sentinel dosing approach should be adopted, accompanied by a cautious dose escalation design. This design aims to determine the pharmacokinetic window that facilitates the clearance of pathogenic cells without inducing excessive systemic inflammation.^27^^,^^59^^,^^67^ If feasible, the trial must incorporate uniform immune monitoring, including quantitative CAR cell kinetics, high-resolution cytokine combinations, anti-vector and anti-CAR antibody detection, and tissue sampling. These measures are essential to capture targeted effects, such as B cell dynamics and autoantibody levels, along with the early safety signals of cytokine release syndrome and immune effector cell-associated neurotoxicity syndrome.^77^ Adaptive designs that permit adjustments to the dosing interval or route of administration during a trial can expedite the identification of treatment regimens that balance efficacy and tolerability. Additionally, biomarkers such as rapid B-cell clearance and a decline in pathogen antibody levels can provide early feedback to guide dose escalation decisions.^78^ Given that a temporary vector system may necessitate repeated dosing, early studies should prospectively assess the immunogenicity of the delivery vector and the feasibility of repeated dosing rather than assuming that a single dose will confer lasting effects (Table 2).

**Table 2:** Published clinical evidence of CAR-T therapy in autoimmune diseases.

Disease	Intervention	Primary outcome	Reference
Refractory systemic lupus erythematosus	Autologous anti-CD19 CAR-T (second-generation constructs) given as a single infusion after lymphodepletion	Safety/feasibility; clinical remission by DORIS criteria and major reductions in SLEDAI; profound B-cell depletion and normalization of autoantibodies (primary endpoints reported as safety and disease activity measures).	84
Systemic lupus erythematosus, systemic sclerosis, idiopathic inflammatory myositis	Autologous CD19 CAR-T (clinical case series/monocentric program & CASTLE trial patients)	Safety and clinical efficacy endpoints: proportion achieving drug-free remission (DORIS/disease-specific response) and safety (cytokine release syndrome, infections). Reported sustained remissions in multiple patients.	12
Generalized myasthenia gravis	Autologous RNA-engineered CAR-T (rCAR-T; Descartes-08; repeated RNA dosing, no lymphodepletion in many patients)	Safety/tolerability in phase 1b/2a; secondary: clinical activity measures (MG-ADL, QMG)—study reported no dose-limiting toxicities/cytokine release syndrome/immune effector cell-associated neurotoxicity syndrome and clinically meaningful reductions in myasthenia gravis severity scores.	85
Refractory biopsy-confirmed lupus nephritis	Autologous anti-BCMA CAR-T (single infusion after lymphodepletion)	Reported deep peripheral B-cell depletion, clinical responses, and limited cytokine release syndrome.	86
Severe myositis & Systemic sclerosis	Healthy donor-derived CD19 CAR-T cells engineered via CRISPR-Cas9 (Allogeneic anti-CD19 CAR-T)	Complete B-cell depletion by 2 weeks; major improvement in clinical response indices at 6 months; no serious cytokine release syndrome or adverse events reported	13
Refractory systemic lupus erythematosus	*In vivo* CD19 CAR-T (LNP-mRNA)	Safety and B-cell depletion; successful *in vivo* generation of CD19 CAR-T cells, rapid B-cell clearance, clinical improvement	67

## Comparison with alternative CAR-based strategies

Beyond conventional cytotoxic CAR-T strategies that primarily deplete broad B-cell populations, several next-generation CAR platforms have emerged to redefine the re-establishment of immune tolerance in autoimmune diseases. CAR-Tregs are designed to deliver antigen-specific immunosuppression rather than immune ablation, combining CAR-guided tissue targeting with suppressive programs of forkhead box P3-positive (FOXP3^+^) Tregs to locally restrain pathogenic immune responses while preserving systemic immunity. Preclinical and translational studies have suggested that CAR-Tregs can restore tolerance in organ-specific autoimmune and transplant settings, although challenges remain in maintaining lineage stability and scalable manufacturing.^79^^,^^80^ In parallel, antigen-precision strategies, such as CAAR-T and T-cell antigen coupler (TAC-T) designs, directly address the specificity dilemma inherent in pan-B-cell targeting by enabling selective elimination of autoreactive B-cell clones based on their pathogenic B-cell receptor, thereby sparing protective humoral immunity. A landmark demonstration of Dsg3-CAAR-T in pemphigus vulgaris illustrates the feasibility of this concept for autoimmunity.^6^^,^^81^ Furthermore, allogeneic “off-the-shelf” CAR-T platforms offer a complementary solution to the accessibility and manufacturing bottlenecks of autologous *ex vivo* CAR-T cells by enabling standardized, rapid-delivery products, although host-versus-graft rejection and durability without prolonged immunosuppression remain major translational barriers. Advances in multiplex gene editing, human leukocyte antigen (HLA) engineering, and immune cloaking strategies are progressively mitigating these limitations and expanding the feasibility of allogeneic CAR-T cells for non-malignant diseases.^13^^,^^25^

## Manufacturing and practical application situation

The manufacturability and cost of the product will determine whether *in vivo* CAR-T therapy can progress beyond the proof-of-concept stage to become a widely applicable treatment for autoimmune diseases, rather than remain a specialized option limited to a few tertiary hospitals. The LNP-based platform benefits from the rapid industrial-scale experience gained during the deployment of mRNA vaccines, which includes an established GMP lipid supply chain, scalable mRNA synthesis, and simplified low-temperature cold chain logistics.^56^ In contrast, the viral platform leverages existing viral vector manufacturing technology, but encounters higher complexity per batch and regulatory reviews, leading to increased costs.^69^ Strategically, the development path aimed at achieving immune reset and limited dosing can provide long-lasting clinical benefits while reducing hospital resource utilization and total costs compared with chronic biological therapies. Auxiliary methods designed to avoid generating protective immune responses, such as CAAR design targeting antigen-specific autoreactive B cells or CAR-Treg strategies that actively re-establish tolerance, are anticipated to offer higher treatment specificity and improved long-term safety.^82^^,^^83^ When the antigen target is well-defined, these precise strategies should be prioritized.

Together, these coordinated developments outline a coherent translational trajectory, transforming *in vivo* CAR-T cells from an experimental concept into a precise, controllable, and durable therapeutic modality for autoimmune diseases.

## Discussion

*In vivo* CAR-T therapy represents a significant advancement in the application of cell-based immunotherapy for autoimmune diseases. This advancement is realized by substituting the intricate *ex vivo* manufacturing process with the direct reprogramming of the patient's own lymphocytes. Such a transformation not only offers transformative potential but also imposes clear responsibilities. Experimental investigations of viral and non-viral delivery platforms have demonstrated that modified constructs can be effectively introduced into endogenous T cells, resulting in the generation of effector cells capable of identifying and eliminating pathogenic immune sites. Preliminary results from human trials suggest that the biological activity of these cells correlates with clinical improvements in various diseases. The promise of this approach lies in its ability to precisely target autoreactive B cells, plasma cells, or pathogenic antigen-presenting cells, achieving an immune reset that a few persistent biological agents can accomplish, while also simplifying the manufacturing process, thereby enhancing accessibility.

Several interrelated challenges must be addressed to achieve this goal. First, safety should always be a top priority, as uncontrolled cytokine release or neuroinflammation is unacceptable in patients with chronic diseases. Therefore, in the selection of platforms, vector design, dosing strategies, and engineering of control switches, priority should be given to activities characterized by predictability and modularity. In addition, built-in mechanisms should be incorporated to terminate or weaken CAR function in the event of severe toxicity. Second, delivery specificity and biodistribution require continuous engineering improvements to ensure that therapeutic substances reach circulating and tissue-resident lymphocytes without excessive non-targeted transfection into the liver or other organs. This can be achieved through ligand-directed targeting, optimization of lipid or capsid chemistry, and improved delivery routes. Third, the balance between persistence and reversibility must align with the disease biology. Persistent genomic insertion may be suitable for eliminating a single pathogenic clone, whereas transient expression may be more appropriate for diseases requiring temporary clearance and allowing for immune reconstitution. Fourth, a stringent clinical development path must be established, including sentinel doses, adaptive escalation, unified immune monitoring, and the validation of early biomarkers that can capture target cell exhaustion and systemic inflammation. Fifth, manufacturing and economic planning should be integrated as early as possible to ensure that platform selection supports scalable and cost-effective production and distribution, with payment models reflecting realistic expectations of long-term benefits and upfront costs. Finally, scientific innovations should persist in pursuing enhanced specificity through the development of antigen-specific CAAR constructs and engineered regulatory T cells. These advances are aimed at restoring immune tolerance, without inducing widespread immune destruction.

Methodologically, preclinical models should accurately reflect the tissue microenvironment that sustains the chronic autoimmunity. Investment in nonhuman primate studies is crucial for evaluating biodistribution, immunogenicity, and long-term safety. Regulatory participation must be repeated and transparent, with long-term tracking and monitoring of implant sites, consistent with the clinical trial design. Fair access pathways and cautious patient screening criteria are essential to ensure that the most vulnerable patients can benefit while minimizing risks. Collaboration among academic researchers, industry partners, regulatory agencies, and patient groups accelerated this research process. This includes unified testing methods, sharing both positive and negative results, and reaching consensus on acceptable risk thresholds.

If these scientific, clinical, regulatory, and economic aspects can be simultaneously advanced, *in vivo* CAR-T technology may evolve into a practical and precise immunomodulatory tool that provides long-lasting relief for various autoimmune diseases. To achieve this goal, continuous multidisciplinary efforts, rigorous clinical science, and a firm commitment to translate technological advancements into safe and effective therapies for patients currently lacking a cure are required.

## CRediT authorship contribution statement

**Yufan Zhao:** Writing – review & editing, Writing – original draft, Methodology, Investigation, Formal analysis, Data curation. **Yuhang Liu:** Writing – review & editing, Writing – original draft, Methodology. **Xiaochun Peng:** Writing – review & editing, Writing – original draft, Validation, Supervision, Resources. **Xin Ma:** Writing – review & editing, Writing – original draft, Project administration, Conceptualization. **Quanjun Yang:** Writing – review & editing, Writing – original draft, Supervision, Project administration, Funding acquisition, Conceptualization.

## Data availability

The data used in this study are available upon request from the lead contact.

## Funding

This work was supported by grants from the 10.13039/501100001809National Natural Science Foundation of China (No. 82272925) and the Shanghai Jiao Tong University School of Medicine Research Physician Program (China) (No. 20240815).

## Conflict of interests

The authors declared no competing interests.
